# Impact of Germination Time on Resveratrol, Phenolic Acids, and Antioxidant Capacities of Different Varieties of Peanut (*Arachis hypogaea* Linn.) from China

**DOI:** 10.3390/antiox10111714

**Published:** 2021-10-27

**Authors:** Ziying Zhou, Zhili Fan, Maninder Meenu, Baojun Xu

**Affiliations:** Food Science and Technology Program, BNU-HKBU United International College, 2000, Jintong Road, Tangjiawan, Zhuhai 519087, China; z448123456789@163.com (Z.Z.); zhilifan@163.com (Z.F.); meenu_maninder@yahoo.com (M.M.)

**Keywords:** peanut, germination, phenolic compounds, antioxidant capacities, *trans*-resveratrol

## Abstract

In China, peanut sprouts are popular among consumers as functional vegetables. This study reports the change in total phenolic content (TPC), total flavonoid content (TFC), monomeric anthocyanin content (MAC), vitamin C, *trans*-resveratrol content, antioxidant capacities, and phenolic profile of three different varieties of peanut during 8 days of germination. The TPC, TFC, and antioxidant capacity of peanut samples were reduced and then increased with an increase in germination time. TFC values were highly correlated with 2,2-diphenyl-1-picrylhydrazyl (DPPH) and ferric reducing antioxidant power (FRAP) values. MAC values of peanuts were first increased and then decreased during 8 days of germination. The TFC, DPPH, and FRAP values of germinated peanuts were lower compared to the non-germinated peanut. Germination of peanut samples enhanced the total phenolic acids and *trans*-resveratrol content, but the vitamin C content of peanut sprouts was lower than ungerminated peanuts.

## 1. Introduction

Peanut (*Arachis hypogaea* Linn.) is an important legume crop that belongs to the *Fabaceae* family and is widely cultivated in tropics and subtropics [1,2]. China is the largest producer of peanuts followed by India, Nigeria, and Sudan. The overall production share of peanuts by Asian countries is 55.9%, African countries is 34.1% followed by Americas 9.9% [3]. Peanuts are the rich source of protein (22–30%), fat (42–49%), carbohydrate (15–21%), and fibers [2]. Peanuts are mostly reported to exhibit a significant amount of linoleic acid (polyunsaturated fatty acid C18:2) and oleic acid (monounsaturated fatty acid, C18:1) which reduce blood LDL-cholesterol levels, improve blood lipid profile and reduce the incidence of cardiovascular diseases [2]. Peanuts are also regarded as a good source of high-quality protein as it contains all essential amino acids required for normal human growth and metabolism [1,2]. Along with all these macronutrients, peanuts also contain a significant amount of micronutrients such as vitamins, phenolics, flavonoids, and tocopherols which are responsible for antioxidant, antimicrobial, anti-cancer, and anti-inflammatory properties [2,4,5,6,7].

Peanuts are appreciated by consumers of all age groups around the globe due to their unique taste, health benefits, availability, and affordability compared to other nuts. Peanuts are generally consumed as snacks after roasting, drying, and frying. Peanuts are widely used for preparing peanut butter, soup thickener and are also used as a major source of vegetable oils [2,5,8]. Germinated peanuts have also been used in the human diet as a functional food for several centuries. In recent years, peanut sprouts became available in several supermarkets in China as a healthy food [8]. 

During seed hydration and sprouting, several complex biochemical changes occur in seeds [1]. Several factors are reported to affect peanut sprouting, namely cultivation method, time, and temperature. The water cultivation of peanut sprouts improved its ratio of oleic acid and linoleic acid content that is responsible for the reduction in the cholesterol level [9]. Researchers have also reported that the soil culture method may reduce the quality of peanut sprouts since this method had an apparent influence on peanut’s radicle elongation [10]. The temperature was reported as another major factor that exhibits a significant influence on the growth of peanut sprouts. Previous research revealed a significant increase in height and fresh weight of peanut sprouts with an increase in the temperature. In addition, 30 °C was mentioned as an optimum temperature to achieve the best emergence rate for peanuts [11]. 

Peanut sprouts are reported to be a rich source of several phytochemicals, vitamins, minerals, and proteins [12]. These micronutrients and macronutrients are essential for human health. The peanut sprouts are also reported to be a rich source of flavonoids and phenolic compounds such as resveratrol, arachidin-1, and piceatannol which are responsible for several health benefits, such as the prevention of diabetes and various cancers [1,12,13]. These phenolic compounds, secondary metabolites produced in plants, are responsible for antioxidant activity, disease prevention and also exhibit several health-promoting properties [14,15,16]. These compounds also exhibit several biological functions such as anti-inflammatory activity, antiplatelet activity, and estrogenic activity [17,18,19]. In addition, flavonoids and phenolic compounds are also reported to exhibit beneficial effects in the treatment of neurodegenerative diseases and ischemia [19]. Recently, peanut sprout extracts supplementation was reported to improve abdominal obesity and overall health indices of overweight and obese women [13]. In addition, peanut sprout extracts were also mentioned to exhibit neuroprotective activities against the oxidative stress in SK-N-SH cells induced by paraquat [20]. Another study on Kalasin 2 cultivar peanut sprout crude extract revealed high anti-inflammatory effects that were related to its polyphenolic content and antioxidant properties [17].

Previously, the resveratrol contents of peanut sprouts were enhanced approximately five times on day 9 of germination compared to day 1. In addition, sucrose, glucose, and total free amino acid content were increased significantly whereas crude protein content of peanuts was decreased. An extensive degradation in large sodium dodecyl sulfate-polyacrylamide gel electrophoresis protein molecules of peanut sprouts was also observed after 9 days of germination [12]. Another study on the chemical composition of peanut sprouts revealed a significant increase in total phenolics, thiamine, folic acid, proline, methionine, aspartic acid, minerals, and water content followed by short-term germination [8]. Researchers have found the highest phenolic content (40.67 ± 2.62 μg gallic acid/g DW), DPPH free radical scavenging activity (DPPH) (80.51 ± 1.47 mmol Trolox/g DW), and ferric reducing antioxidant capacity (FRAP) (171.33 ± 8.59 mmol ascorbic acid/g DW) in the Kalasin1 cultivar of peanut after three days of germination. Whereas the highest resveratrol content (6.44 ± 1.26 μg/g DW) was observed in Kalasin 2 sprouts on the second day of germination [1]. The available studies have explored either total phenolics, antioxidant activity using a particular assay, or certain bioactive compounds. However, no report is available on the detailed study of phytochemical compositions and antioxidant profiles of different varieties of peanut during germination. Thus, this study was conducted with an objective to study the change in moisture content, total phenolic content, total flavonoid content, monomeric anthocyanin content (MAC), vitamin C content, *trans*-resveratrol, individual phenolic acid compounds, and antioxidant activities assessed by employing ferric reducing antioxidant power (FRAP) assay, 2-diphenyl-1-picrylhydrazyl (DPPH) assay and 2,2′-azino-bis (3-ethylbenzothiazoline-6-sulfonic acid) (ABTS) assay of different varieties of peanuts collected from China at 0, 2, 4, 6 and 8 day of germination.

## 2. Materials and Methods

### 2.1. Peanut Samples

**Figure 1:**
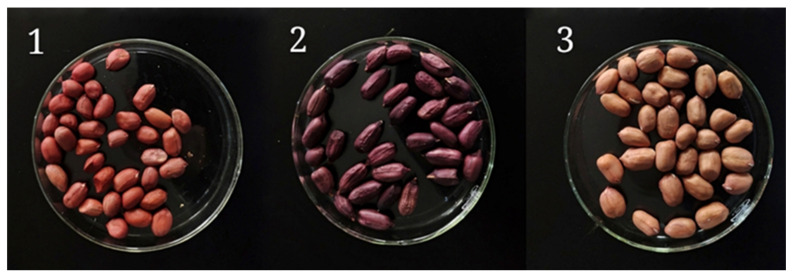
Samples of peanuts from different varieties were used in the study. 1. Silihong, 2. Silihei, 3. Xiaobaisha.

Three different peanut varieties from Shandong province, China were explored in this study. Details regarding the name of variety, type or physical appearance of peanut variety, year of cropping, and source are mentioned in Table S1 and Figure 1. The peanut samples of all these three varieties were cleaned to remove any broken and crushed peanuts. The peanut samples were stored at 4 °C in dark until further analysis.

### 2.2. Chemical Reagents

2-Diphenyl-1-picrylhydrazyl (DPPH), 2,2′-azino-bis (3-ethylbenzothiazoline-6-sulfonic acid) (ABTS), (+)-catechin, 2,4,6-tri(2-pyridyl)-s-triazine (TPTZ), 2, 6-dichloroindophenol, *L*-ascorbic acid were procured from Shanghai Yuanye Biological Technology Co., Ltd. (Shanghai, China). Folin–Ciocalteu reagent was purchased from Shanghai Sanjie Biotechnology Co., Ltd. (Shanghai, China). Sodium carbonate, acetonitrile, methanol (high-performance chromatography grade), methanol (analytical grade), acetic acid, and trifluoroacetic acid (TFA) was supplied by Tianjin Nuoke Technology Development Co., Ltd. (Tianjin, China). Whereas, meta-phosphoric acid, citric acid, and sodium hydroxide (NaOH) were purchased from Damao Chemical Reagent Co., Ltd. (Tianjin, China). 

### 2.3. Germination and Sample Preparation

The peanut seeds were germinated in the dark according to a previously described method [21]. Briefly, the peanut samples were soaked in tap water for 24 h, followed by spreading on a gauze-covered plate. The gauze was watered twice a day. After 2 days, the germinated peanut seeds were transferred into the seedling raising plates filled with water. The seeding-raising plates were put into a seed germinator at 30 °C (Model: FYZ-280, Zhejiang Jiangnan Instrument Factory, Ningbo, China). The water in seedling raising plates was changed twice a day. The peanut sprout samples were obtained at days 0, 2, 4, 6, and 8, respectively. After harvesting, the germinated peanut samples from each group were ground for 1 min using a blender (XBLL-25, Shanghai Shuaijia Electronic Technology Co., Ltd., Shanghai, China). Finally, the ground samples were freeze-dried using a freeze-dryer (Labconco Corporation, Kansas City, MO, USA) and stored at −80 °C until further analysis.

### 2.4. Determination of Moisture Content

The moisture content of peanut samples (3 g) was determined by employing a rapid moisture analyzer (LHS20-HR, Shanghai Tianmei Tianping Instrument Co., Ltd., Shanghai, China) in triplicate.

### 2.5. Determination of Total Phenolic Content (TPC)

All the peanut samples were extracted according to a previously described method [22]. Briefly, 0.5 g of peanut sample was extracted twice with 5 mL of extraction solvent (acetone/water/acetic acid, 70:29.5:0.5, *v*/*v*/*v*). For extraction, samples were shaken for 4 h followed by incubation at room temperature in dark for 16 h followed by centrifugation at 6000 rpm for 2 min. The resultant extract was stored at 4 °C for further analysis.

Total phenolic content (TPC) of peanut samples was determined using Folin-Ciocalteu assay [23]. Gallic acid was applied as an external standard for TPC determination. Briefly, 50 μL of sample extract was mixed with distilled water (3 mL), Folin-Ciocalteu reagent (250 μL), and of 7% Na_2_CO_3_ solution (750 μL). The absorbance of the resultant reaction mixture was observed at 765 nm by a UV–visible spectrophotometer (UT-1901, Beijing Purkinje General Instrument Co., Ltd., Beijing, China) after 1 h of incubation in dark. The TPC values of peanut samples were expressed as milligram gallic acid equivalents per gram of dried sample (mg GAE/g DW).

### 2.6. Determination of Total Flavonoid Content (TFC)

TFC content of peanut samples was determined by employing the aluminum chloride colorimetric method [18]. Briefly, sample extract (250 μL) was mixed with distilled water (1250 μL), followed by the addition of 5% Na_2_NO_3_ solution (75 μL). After 6 min, 10% AlCl_3_·6H_2_O (150 μL) was added to the reaction mixture. After 5 min, 500 μL of 1 M NaOH and 275 μL of distilled water were added. The absorbance of the reaction mixture was measured at 510 nm. The TFC values of peanut samples were expressed as mg of (+)-catechin equivalents per g of dried sample (mg CAE/g DW).

### 2.7. Determination of Monomeric Anthocyanin Content (MAC)

MAC values of peanut samples were determined by employing a previously mentioned pH differential method [24]. The peanut sample extracts were diluted with pH 1.0 buffer and with pH 4.5 buffer. After 15 min, the absorbance of reaction mixtures was measured using a UV-Vis spectrophotometer at both 700 nm and 510 nm. The anthocyanin pigment concentration of peanut samples was expressed as cyanidin-3-glucoside equivalents (*w*/*w*%).

### 2.8. Determination of DPPH Free Radical Scavenging Activity (DPPH)

DPPH value of peanut samples was determined by employing a previously described colorimetric method using Trolox as a standard [23]. The peanut sample extract (0.2 mL) was mixed with DPPH reagent (3.8 mL) and vortexed. The reaction mixture was incubated for 30 min in dark at room temperature. The absorbance of the resultant reaction mixture was recorded at 517 nm and results were expressed as Trolox equivalents per g of dried samples (μmol TE/g DW).

### 2.9. Determination of ABTS Free Radical Scavenging Activity (ABTS)

ABTS free radical scavenging activity of peanut samples was accessed by employing a previously described method [25]. Trolox was used as an external standard for the determination of ABTS values of samples under investigation. Briefly, the sample extracts (20 μL) were mixed with ABTS reagent (1 mL) and the absorbance of the resultant reaction mixture was recorded at 734 nm against an ethanol blank. The results were expressed as μmol of Trolox equivalents per gram of the dried samples (μmol TE/g DW).

### 2.10. Determination of Ferric Reducing Antioxidant Capacity (FRAP)

Ferric reducing antioxidant capacity (FRAP) of sample extracts was determined by using a previously reported colorimetric assay [26]. FeSO_4_ was employed as an external standard. Briefly, 100 μL of sample extracts were mixed with 300 μL distilled water and FRAP reagent (acetate buffer, TPTZ, FeCl_3_ solution, and distilled water). After 4 min, the absorbance of the reaction mixture was recorded at 593 nm and FRAP values of peanut samples were expressed as mmol of Fe^2+^ equivalent (FE^2+^) per 100 g of dried sample (mmol FE^2+^/100 g DW).

### 2.11. Determination of Vitamin C Content

The peanut samples were mixed with 8 mL of 3% metaphosphoric acid and stirred at high speed for 2 min using a turbo mixer followed by centrifugation at 8500 rpm for 5 min. The supernatant was collected in 50 mL volumetric flasks. The buffer extract was diluted to 1/5 of the original concentration using citrate phosphate buffer. Then, after the addition of 2.5 mL of indoxyl solution, the absorbance of the reaction mixture was directly observed by the spectrophotometer (Shanghai Jinke Electronic Technology Co., Ltd., Shanghai, China). The excess of ascorbic acid was then added to complete discoloration of the dye, again as observed with the spectrophotometer, which was regarded as the blank. Finally, the vitamin C content was obtained by subtracting the blank value from the absorbance read at 520 nm and comparing it with the standard curve [27]. 

### 2.12. Determination of Trans-Resveratrol

The *trans*-resveratrol content of peanut samples was determined according to a previously mentioned procedure [28]. The sample was extracted with 80% ethanol in an ultrasonic bath for two hours. The sample was filtered to collect supernatant followed by vacuum drying. The dried supernatant was redissolved in 3 mL of acetonitrile and then filtered through a 3 µm syringe filter into an HPLC (High-Performance Liquid Chromatography) vial.

The HPLC analysis of resultant extract was performed using RP-HPLC system (Waters Associates, Milford, MA, USA) equipped with a C18 column (Zorbax Stablebond Analytical SB-C18 column, 4.6 mm × 250 mm, 5 μm). The ultrapure water containing 0.05% acetic acid was employed as mobile phase A and HPLC-grade acetonitrile was used as mobile phase B. An isocratic elution with mobile phase A 78% and B 22% was employed at a flow rate of 0.9 mL/min for 12 min at room temperature. The column temperature was set at 30°C, sample injection volume was 10 µL and the wavelength of the detector was recorded at 306 nm. The *trans*-resveratrol contents were expressed as micrograms of phenolic acid per gram of dried sample (ng/g DW).

### 2.13. Determination of Phenolic Acid 

Briefly, the peanut sample was extracted twice with 10 mL of extraction solvent (methanol/water/acetic acid/butylated hydroxytoluene (BHT) = 85:15:0.5:0.2) by shaking at 300 rpm for 4 h at room temperature followed by centrifugation at 6000 rpm for 20 min. After centrifugation, the supernatant was filtered through Whatman#1 filter paper and concentrated at 45 °C under vacuum to remove the extraction solvent. Then, the dried residue was re-dissolved in 2.5 mL of 25% methanol. Finally, this methanol sample solution was filtered through a 0.2 µm PVDF syringe filter in an HPLC vial.

The quantitative analysis of phenolic acids was performed by employing HPLC according to a previously described method [22]. A Waters Associates (Milford, MA, USA) chromatography system equipped with a model 418 LC spectrophotometer, a model 720 system controller, a model 7125 loading sample injector, and a model 6000A solvent delivery system was used. A Zorbax Stablebond Analytical SB-C18 column (4.6 mm × 250 mm, 5 μm) from Agilent Technologies, Rising Sun, MD, USA at 40 °C was used for separation of phenolic acids. Twenty microliters of the sample extract were employed, and the detector was set at 270 nm.

A linear elution gradient of 0.1% TFA (solvent A) and methanol (solvent B) was employed. Solvent A was decreased from 95% at 0 min to 0% at 76 min at a flow rate of 0.7 mL/min and the sample injection volume was 20 μL. The stock solution of phenolic acids mixture was prepared in 25% methanol followed by the dilution to appropriate concentrations. The calibration curves of phenolic acids were obtained by plotting different concentrations of a particular phenolic acid versus corresponding peak areas. The phenolic acids in the peanut samples were identified by comparing their retention time and UV spectra with the retention time and UV spectra of standard phenolic acid peaks. The phenolic acid contents were expressed as micrograms of phenolic acid per gram of dried sample (μg/g DW).

### 2.14. Statistical Analysis

All experiments were conducted in triplicates and the data were presented as mean ± standard deviation. The significant differences (*p* < 0.05) among the mean values of different samples were analyzed by performing the Duncan test using IBM SPSS Statistics version 25 (IBM Corporation, New York, NY, USA). 

## 3. Results

### 3.1. Radicle Length of Different Varieties of Peanuts during Germination

**Figure 2:**
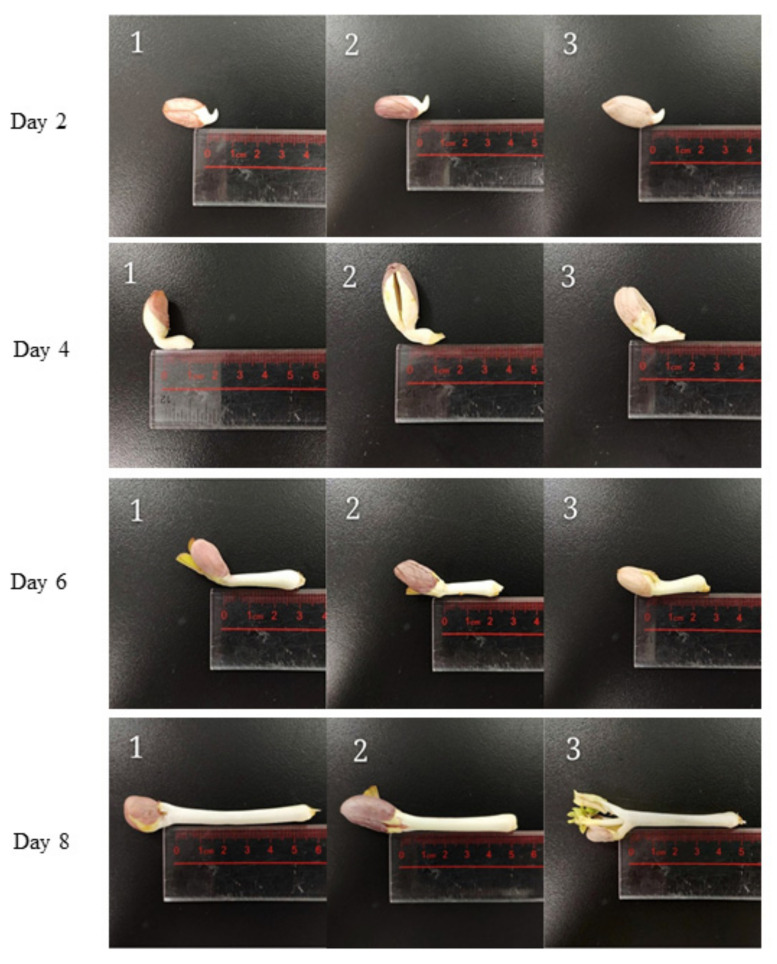
Development and length of the radicle of different varieties of peanuts of different varieties namely, (1) Silihong, (2) Silihei, and (3) Xiaobaisha during germination.

The changes in radicle length of peanuts at days 0, 2, 4, 6, and 8 are presented in Figure 2. The three kinds of peanuts investigated in the present study followed a similar growth trend (Figure 2 and Figure S1). Peanut Silihong grows slightly faster than the other two kinds of peanut. The length of radicle in the case of peanut Silihong reached 5.3 cm on day 8 while the length of peanut Silihei and Xiaobaisha was measured as 5.0 cm and 5.2 cm, respectively. A rapid increase in the radicle length was observed from day 4 to day 6 of germination. The elongation rate for Silihong, Silihei, and Xiaobaisha was observed to be 200%, 170%, and 136%, respectively. It was also observed that on germination days 6 to 8, the elongation rate was 77%, 85%, and 100% for Silihong, Silihei, and Xiaobaisha, respectively which was significantly lower compared to as observed on days 4 to 6.

### 3.2. Moisture Content of Different Varieties of Peanuts during Germination

The data related to the moisture content of three different varieties of peanut is presented in Table S2. The moisture content of the peanut samples was found to be increased with an increase in germination time. The peanut variety Silihong exhibited a higher moisture content compared to the other two peanut varieties. A rapid increase in moisture content was observed in the case of Silihong (309%), Silihei (360%), and Xiaobaisha (294%) from day 0 to day 2 of germination. However, the increase in moisture content of peanut samples was slightly lower from day 2 of germination to day 8. The highest increase in the moisture content during this period was observed in the case of Silihong (36%) during days 2 to 4. Whereas the highest increment in the moisture content in the Silihei variety was 32% from day 4 to 6 and 26% in the case of variety Xiaobaisha from day 6 of germination to day 8.

### 3.3. Total Phenolic Content (TPC) of Different Varieties of Peanuts during Germination

**Figure 3:**
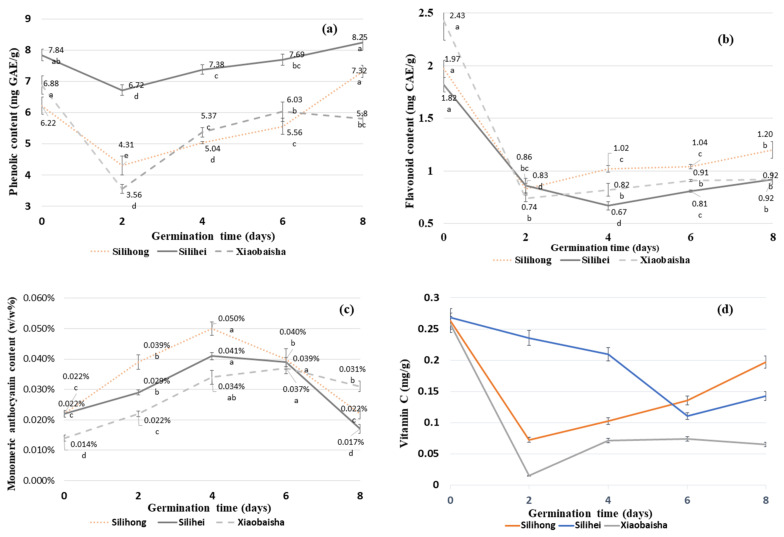
Kinetic changes (**a**) TPC, (**b**) TFC, (**c**) MAC, (**d**) vitamin C of peanut during germination. Data marked with the same letters were not statistically significant (*p* > 0.05).

TPC values for three different kinds of peanut from day 0 to day 8 of the germination are presented in Figure 3a. The peanut variety Silihei exhibits relatively high values of TPC from day 0 to day 8 compared to the peanut variety Silihong and Xiaobaisha. The TPC values for peanut variety Silihei was 7.84 mg GAE/g DW, while Silihong and Xiaobaisha contain 6.22 and 6.88 mg GAE/g DW, respectively on day 0. From days 0 to 2, a significant decrease in the TPC values was observed, especially in the case of peanut variety Xiaobaisha, where the TPC value was reduced from 6.88 to 3.56 mg GAE/g DW. However, from day 2 of germination to day 8, an increasing trend in the TPC values of all peanut varieties was observed except in the case of peanut variety Xiaobaisha that exhibited 6.03 mg GAE/g DW of TPC on day 6 of germination and 5.8 mg GAE/g DW of TPC on day 8 of the germination.

The highest increment in the TPC values of three different varieties of peanuts was observed at different stages of germination. In the case of peanut variety Silihong, the maximum increase in the TPC value (31.7%) was observed from day 6 of germination to day 8. However, in the case of peanut variety Xiaobaisha, the maximum increase in the TPC value (50.8%) was observed from day 2 of germination to day 4 of germination. The increasing trend of TPC value of peanut variety Silihei was relatively stable and the maximum increase in TPC value (9.8%) was observed from day 2 to day 4 of germination. Overall, the TPC values of peanut variety Silihong and Silihei on day 8 of germination were higher compared to the non-germinated peanuts and the percentage increment in case of variety Silihong and Silihei on day 8 was observed to be about 17.7% and 5.2%.

### 3.4. Total Flavonoid Content (TFC) of Different Varieties of Peanuts during Germination

The TFC values for three varieties of peanut are presented in Error! Reference source not found. It was observed that the peanut variety Xiaobaisha exhibit the highest value for TFC (2.43 mg CAE/g DW) on day 0 followed by peanut variety Silihong (1.97 mg CAE/g DW) and Silihei (1.82 mg CAE/g DW). A significant decrease in the TFC values of all three varieties of peanuts was observed from day 0 to day 2 of germination. The maximum decrease in the TFC value (228.3%) was observed in the case of peanut variety Xiaobaisha on day 2 of germination followed by peanut variety Silihei (171.6%) and Silihong (137.3%) from day 0 to day 4 of germination. Furthermore, an increment in TFC values of peanut variety Xiaobaisha and Silihong was observed on day 4 of germination, however, peanut variety Silihei presented a decrease in the TFC value on day 4. 

The TFC values of germinated peanut samples were less compared to the non-germinated samples and the decreasing percentage for peanut variety Silihong, Silihei, and Xiaobaisha from day 0 to day 8 was observed to be 64.2%, 97.8%, and 164.1%, respectively.

### 3.5. Monomeric Anthocyanin Content (MAC) of Different Varieties of Peanuts during Germination 

The MAC value of three varieties of peanut during germination is presented in Error! Reference source not found. A significant increase in the MAC values of all varieties of peanuts was observed from day 0 to day 4 of the germination followed by a decrease in MAC values from 4 to day 8 of germination. Overall, the increasing MAC value trend in all peanut varieties is similar. However, from day 4 to 8, the decreasing trend of MAC value is different in all three varieties. On day 6, all the three peanut varieties presented in significantly different values for MAC, and on day 8 minimum decrease in the MAC value was presented by peanut variety Xiaobaisha followed by Silihong and Silihei.

The highest values of MAC for Silihong (0.050%), Silihei (0.041%), and Xiaobaisha (0.037%) were observed on day 4, day 4, and day 6 of germination, respectively. The maximum increase percentage of MAC values for Silihong (127.3%), Silihei (86.4%), and Xiaobaisha (164.3%) were on day 4 or 6 of germination compared to the non-germinated samples.

### 3.6. DPPH Free Radical Scavenging Activity (DPPH) of Different Varieties of Peanuts during Germination

**Figure 4:**
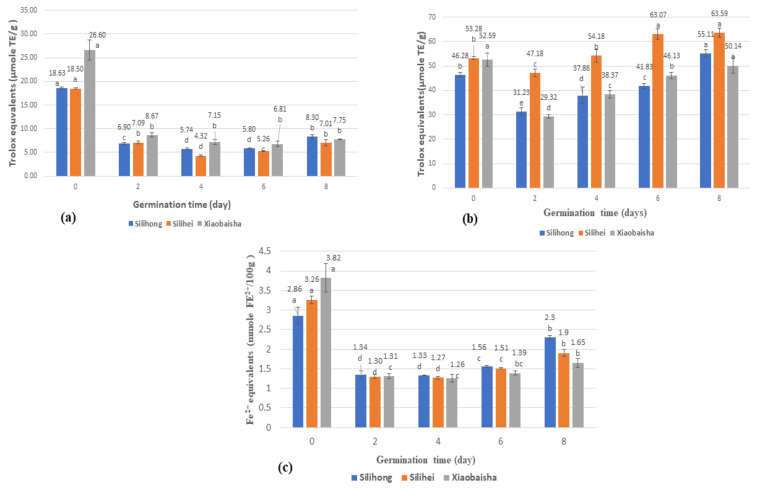
Kinetic changes of (**a**) DPPH free radical scavenging activity, (**b**) ABTS radical scavenging activity, (**c**) ferric reducing antioxidant capacity (FRAP) of peanut during germination. Data marked with the same letters were not statistically significant (*p* > 0.05).

The change in the DPPH values of peanuts of three different varieties from day 0 to day 8 of germination is presented in Figure 4. Overall, a sharp decrease in the DPPH value of peanut samples was observed initially on day 2. However, on day 8 of germination, a slight increase in DPPH values was observed and the percentage increase in DPPH value was observed to be 43.1%, 33.3%, and 13.8% for Silihong, Silihei, and Xiaobaisha, respectively. On day 0, peanut variety Xiaobaisha had the highest value for DPPH assay (26.60 μmol TE/g DW) followed by Silihong (18.63 μmol TE/g DW) and Silihei (18.50 μmol TE/g DW). It was noted that the DPPH values of peanut variety Xiaobaisha decreased until day 6 and increased on day 8 of germination. However, the DPPH value of Silihong and Silihei was increased from day 6 to day 8. On the 8^th^ day of germination, the highest DPPH value was found in the peanut variety Silihong (8.30 μmol TE/g DW) followed by Xiaobaisha (7.75 μmol TE/g DW) and Silihei (7.01 μmol TE/g DW). Overall, the germinated peanut samples of all varieties presented a lower DPPH value compared to the raw peanut samples and the decreasing percentage from day 0 to day 8 was about 124.5% for Silihong, 163.9% for Silihei, and 243.2% for Xiaobaisha.

### 3.7. ABTS Free Radical Scavenging Activity (ABTS) of Different Varieties of Peanuts during Germination

The ABTS free radical scavenging activity for different varieties of peanuts from day 0 to day 8 of germination was presented in Figure 4b. Initially, an abrupt decrease in ABTS values of all peanut samples was observed from day 0 to day 2 followed by an increase in ABTS values from day 2 to day 8 of germination. From day 2 to day 8 of germination, the ABTS value of peanut variety Silihei was observed to be significantly higher compared to peanut varieties Silihong and Xiaobaisha. On the 8^th^ day of germination, the peanut variety Silihong (55.11 μmol TE/g DW) and Silihei (63.59 μmol TE/g DW) presented higher values for ABTS compared to day 0, which were 46.28 μmol TE/g DW for Silihong and 53.28 μmol TE/g DW for Silihei and the percentage increase on day 8 was 19.1% for Silihong and 18.2% for Silihei compared with day 0. Unlike Silihong and Silihei, the ABTS value of Xiaobaisha on the last germinated day (50.14 μmol TE/g DW) was slightly lower than the non-germinated peanut sample (52.59 μmol TE/g DW).

The highest percentage increase in the ABTS values for three different varieties of peanuts was observed at different germination times. The highest percentage increase in peanut variety Silihong was observed on day 6 to day 8 (31.7%), whereas for peanut variety Silihei, the highest percentage increase was observed on days 4 to 6 (16.4%) and in case of Xiaobaisha, the highest percentage increase was observed on day 2 to 4 (30.9%).

### 3.8. Ferric Reducing Antioxidant Capacity (FRAP) of Different Varieties of Peanuts during Germination

The FRAP values for non-germinated and germinated peanut samples of three different varieties were presented in Figure 4c. The FRAP values for all varieties of peanuts were decreased from day 0 to 4 and further increased from day 4 to day 8 of germination. At the beginning of germination, peanut variety Xiaobaisha presented the highest value for FRAP assay (3.82 mmol Fe^2+^/100g DW) while on the last day of germination, its FRAP value (1.65 mmol Fe^2+^/100g DW) was observed to be lower than the FRAP values of peanut variety Silihong (2.3 mmol Fe^2+^/100g DW) and Silihei (1.9 mmol Fe^2+^/100g DW). Overall, the lowest FRAP values were observed on day 4 with Silihong presenting 1.33 mmol Fe^2+^/100g DW, Silihei presenting 1.27 mmol Fe^2+^/100g DW and Xiaobaisha exhibiting 1.26 mmol Fe^2+^/100g DW.

The maximum percentage decrease was observed from day 0 to day 2 of germination to be 112.7% for Silihong, 150.8% for Silihei, and 191.6% for Xiaobaisha. All peanut samples had a rapid decline and then a slight increase in FRAP values during the whole period of germination. The maximum increase in the FRAP values of three varieties of peanut was observed on day 8, and the percentage increase was 42.2% in the case of Silihong, 49.6% in the case of Silihei, and 40.0% in the case of Xiaobaisha compared with day 4 of germination. However, the FRAP values on day 8 were significantly lower compared to the non-germinated peanut sample, and the percentage decrease for Silihong, Silihei, and Xiaobaisha was observed to be 24.3%, 71.6%, and 131.5%, respectively. 

### 3.9. Vitamin C Content of Different Varieties of Peanuts during Germination 

As shown in Figure 3d, the vitamin C content of three types of peanuts follows the same trend. Initially, a significant decrease in vitamin C was observed followed by an increase in vitamin C content from day 6 to day 8 of germination. On the 8^th^ day of germination, the vitamin C content of peanut variety Silihong was 0.197 mg/g DW, whereas the vitamin C content of Silihei was 0.142 mg/g DW and Xiaobaisha was 0.065 mg/g DW. During the first two days of germination, the vitamin C content of all three varieties of peanuts was decreased. Among them, Xiaobaisha variety presented a sharp decline in vitamin C (0.015 mg/g DW) followed by Silihong (0.073 mg/g DW). However, in the case of peanut variety Silihei, the decrease in vitamin C content was quite low and continued until the 6^th^ day of germination (0.111 mg/g DW). From the 4^th^ to the 8^th^ day, peanut variety Silihong presented the highest increase (46%) in vitamin C followed by Silihei and Xiaobaisha. It was interesting to note that the vitamin C content of peanut variety Xiaobaisha was almost stable at 0.07 mg/g DW after day 2 of germination.

### 3.10. Trans-Resveratrol of Different Varieties of Peanuts during Germination

The change in the *trans*-resveratrol content of different varieties of peanuts from day 0 to day 8 of the germination is presented in Table 1. Figure S2a,b present the standard curve of *trans*-resveratrol and typical peanut sample. All three peanut varieties had an overall increasing trend in the *trans*-resveratrol content from day 0 to day 8 of the germination. Table 1 reports the peanut variety, Silihei had higher values for *trans*-resveratrol content. On the 8th day of germination, peanut variety Silihei had 631 ng/g DW of *trans*-resveratrol that was higher compared to the *trans*-resveratrol content of peanut variety Silihong (415.93 ng/g DW) and Xiaobaisha (194.90 ng/g DW). The *trans*-resveratrol content of peanut variety Silihei has increased abruptly during germination with a growth percentage of 3070%. In the case of peanut variety Silihong and Xiaobaisha, a significant increment in the *trans*-resveratrol content was observed from day 2 to day 4 of the germination with an increasing percentage of 298.85% and 129.34%, respectively. The *trans*-resveratrol content of peanut variety Silihong and Xiaobaisha was further decreased from day 6 to day 8 of germination by 222.58 ng/g DW and 86.84 ng/g DW, respectively.

### 3.11. Phenolic acid Profile of Different Varieties of Peanuts during Germination

Ten different phenolic acids were quantified in three different varieties of peanuts during germination (Figure S2c,d). Gallic acid was sharply increased in Silihong from day 0 to day 2 of germination from around 4.58 μg/g DW to around 32.15 μg/g DW, and then at days 4 and 6, gallic acid content was 25.54 and 28.99 μg/g DW and a slight increase in gallic acid (38.44 μg/g DW) were further observed at day 8 (Table 2). However, in the case of Silihei and Xiaobaisha, gallic acid content varied slightly at day 0 to 4 of germination and increased sharply from day 6 to day 8 of germination. The gallic acid content of Silihei was varied from 13.95 to 38.44 μg/g DW, while the gallic acid concentration was varied from 21.14 to 39.78 μg/g DW in the case of Xiaobaisha. The protocatechuic acid in all varieties of peanuts followed the same trend. A slight variation in protocatechuic acid concentration was observed from day 0 to day 4 of germination followed by a rapid increase on day 6 of germination. Overall, the Silihong variety ha trend from 3.72 to 4.01 μg/g DW. An increase in the protocatechualdehyde content was observed in the case of Xiaobaisha variety from 0.91 to 1.95 μg/g DW. On the contrary, the protocatechualdehyde content was reduced from 4.05 to 0.71 μg/g DW in the case of peanut variety Silihei. The *p*-hydroxybenzoic acid of three varieties of peanuts under investigation presented an increasing trend during germination. 

From day 0 to day 6 of germination, gentisic acid of the Silihong variety was decreased from 23.95 to 10.56 μg/g DW, however, a slight increase (11.01 μg/g) was observed on day 8 (Table 2). In contrast, a continuous increase in the gentisic acid level was observed in the case of Silihei and Xiaobaishao except on day 4 of germination. A significant increase in chlorogenic acid was observed in the case of all three varieties under investigation. However, for Silihei, the chlorogenic acid was decreased significantly (7.29 μg/g) from day 2 to day 4 of germination. The syringic acid content of all the peanut varieties was higher on day 0. However, a significant decrease in the syringic acid was observed with an increase in the germination time of all the varieties. The *p*-coumaric acid and syringaldehyde of all varieties were increased from days 0 to 8. In the case of Silihong, the *p*-coumaric acid and syringaldehyde were increased continuously during the whole duration of germination from 6.82 to 38.73 μg/g DW. However, significant variations in *p*-coumaric acid and syringaldehyde content were observed in the case of Silihei and Xiaobaisha from day 0 to day 8 of the germination. The ferulic acid content of all the peanut varieties followed almost a similar trend during germination. In all the peanut varieties, the ferulic acid content was increased steadily from day 2 of germination and then decreased on days 6, 8, and 6 of germination, respectively. In the case of Xiaobaisha, the sinapic acid content was increased from 15.46 to 32.58 μg/g DW from day 0 to day 8. However, in the case of Silihei, the sinapic acid content was varied from 5.55 to 12.07 μg/g DW from day 0 to day 6 and in the case of Silihong, its values varied from 4.55 to 6.90 μg/g DW from day 6 to day 8 of germination. 

## 4. Discussion

### 4.1. Effects of Germination Time on Radicles Length of Different Varieties of Peanuts

As shown in Figure 2 and Figure S1, it was observed that the length of peanut radicle is increased slowly from day 2 to 4, as well as from day 6 to 8. However, a rapid increase in peanut radicle was observed from day 4 to day 6 of germination. This observation is in concordance with a previous study that mentions the elongation rate of radicle length at 30 °C follows a “slow-quick-slow” pattern [29]. In addition, the growth trend followed by peanut radicle in this study was in line with another study, that reported the fastest growth of peanut radicle from day 4 to day 6 of germination at 30 °C [30].

### 4.2. Effects of Germination on the Moisture Content of Different Varieties of Peanuts

According to Table S2, the maximum increase in moisture percentage of peanut samples was observed on day 2 of germination. An increase in the moisture content of peanut samples in the early stage of germination is attributed to the swelling of hydrophilic colloids in peanuts. At this stage, proteins, enzymes, other macromolecules, and organelles in the embryos successively underwent hydration activation [31].

According to a previous study, the increment in the moisture content of seed during the germination process can be divided into three phases namely, swelling water absorption phase, slow water absorption phase, and growth water absorption phase [32]. The percentage variation in the moisture content of peanut samples exhibits a “quick-slow-quick” tendency. As shown in Table S2, a rapid increase in the moisture content of different varieties of peanut samples was observed from day 0 to day 2 of germination. Whereas an increment in the moisture content of peanut samples was slow from day 2 to day 8 as the peanut samples were still in the slow water absorption phase. It was also observed that the moisture content increment is stable from day 2 to day 8 of germination. A similar trend was also reported in a previous study in which the average moisture content for three varieties of peanuts on day 8 of germination was about 83.3% [33].

### 4.3. Effects of Germination on Total Phenolic Content (TPC) of Different Varieties of Peanuts

During germination, TPC values of the three varieties of peanut exhibit an increasing trend. TPC values for peanut varieties Silihong and Silihei were higher on the last day of germination as compared to the non-germinated peanut (Figure 3a). This observation is in complete agreement with a previous study that also mentioned an increase in the TPC values after seed germination [13].

In addition, during days 0 to 2 of germination, the TPC values of all the peanut samples exhibited a decrease. The soaking treatment softened the tissue structure of seeds facilitating the release of polyphenols from cell wall polysaccharides that in turn increase the TPC value. However, the action of polyphenol oxidase led to the degradation of certain free polyphenols by oxidation that further cause a decrease in the TPC value of seed samples [34].

The trend observed in the TPC variation of peanut samples was similar to a previous study. However, the TPC values of non-germinated peanuts in previous reports were different from the TPC value of non-germinated peanut samples. The peanut kernel explored in a previous study was reported to contain 1.53 mg GAE/g of TPC [21]. Whereas, in another study, the TPC value for non-germinated peanut samples was about 0.92 mg GAE/g [35]. However, in the present study, the TPC value of non-germinated peanuts was relatively higher. The reason behind the higher values of TPC in the present study might be due to the variation in the peanut variety and extraction method. The variation in the biochemical composition of a plant sample collected from the different geographical regions is well reported previously [16].

### 4.4. Effects of Germination on Total Flavonoid Content (TFC) of Different Varieties of Peanuts

As shown in Figure 3b, a significant decrease in the TFC values was observed at the beginning of germination followed by a slight increase during the middle of germination. A similar trend in the TFC values of peanuts was also reported previously during germination [21]. The increase in the TFC values of peanuts during germination was previously reported to be due to the synthesis of new flavonoid compounds during the process of germination [36]. The TFC values of peanut samples on the last day of germination were lower than the non-germinated peanut. 

Previous reports also stated that the antioxidant activity of peanuts is mainly due to the presence of colorless pigments mostly flavonoids compared to the colored pigments anthocyanins [4]. Peanuts are the storehouse of several flavonoids such as anthocyanin and catechins namely, epigallocatechin, epicatechin, catechin gallate, and epicatechin gallate. Anthocyanin as well as catechins can dissolve in water. This contributes towards the loss of flavonoids during germination due to water absorption which occurs at that stage. Flavonoids can also be decomposed by light and oxygen. Therefore, in the present study, the lower TFC value observed in the germinated peanut samples was attributed to the water absorption, light, or oxidation during germination [37,38]. Previously, the TFC value of non-germinated peanut was mentioned as 1.0 mg CAE/g [21] that was slightly lower compared to the TFC value of the non-germinated peanut samples in this study. This variation in TFC values among these two studies may be again attributed to different varieties of peanuts investigated and differences in the extraction methods employed.

### 4.5. Effects of Germination on Monomeric Anthocyanin Content (MAC) of Different Varieties of Peanuts

As shown in Figure 3c, the highest MAC values were observed around the 4^th^ day of germination. An increasing trend in the MAC value was observed at the beginning and decreased in the last of germination. From day 0 to day 6 of germination, the MAC values of peanut varieties, Silihong and Silihei were higher compared to the peanut variety Xiaobaisha. The seed coat color of Xiaobaisha was light red whereas the seed coat of Silihong exhibit bright red color and Silihei exhibits a black color that may be the reason behind the lower MAC values of Xiaobaisha compared to the Silihong and Silihei. A previous study on peanuts also mentioned that the samples with darker seed coats exhibit higher values for MAC compared to the peanuts with lighter seed coats [4].

At an early stage of germination, peanut seeds were reported to synthesize anthocyanin with several factors including light, pH, temperature, enzymes, and reactive oxygen species found to be responsible for the reduction of anthocyanins during the mid-point of germination [39]. In addition, anthocyanins are water-soluble flavonoids and several enzymes activated during germination, together with result in a decrease in anthocyanin content due to leaching and oxidation [40]. Also, anthocyanins are sensitive to light and need to be protected from light. According to a previous study, the optimal pH for anthocyanin was about 2 to 3 [41]. Therefore, the higher pH in cultivation water, light, and water absorption was considered responsible for the loss of anthocyanin during germination.

### 4.6. Effects of Germination on Antioxidant Capacities of Different Varieties of Peanuts

As shown in Figure 4, the antioxidant capacities of peanut samples of different varieties present a similar trend during germination. Briefly, a significant decrease in the antioxidant capacities of peanut samples was observed followed by an increment till the 8^th^ day of germination. The antioxidant capacities of peanut samples are contributed mainly by the phenolic and flavonoid compounds present in the seed coat and cotyledon [4]. At the early stage of peanut germination, phenolic compounds in the seed coat responsible for its pigmentation were lost due to soaking and germination, which in turn reduces the antioxidant ability of peanut samples [42]. During the middle of germination, the antioxidant capacities of peanut sprouts start increasing due to the synthesis of phenolic compounds. The findings of the present study are in agreement with a previous study that reported a similar trend in antioxidant capacities of peanut sprout during germination [1].

The DPPH and FRAP values of the germinated peanut samples were lesser on day 8 compared to the non-germinated peanut (Figure 4a,c). The different results of antioxidant capacities determined by employing various assays may be due to the variation in the underlying principle of these assays. The antioxidant capacities of peanut samples assessed by using ABTS assay (Figure 4b) were higher compared to the antioxidant capacity determined by the DPPH assay (Figure 4a). This variation is due to the high stability of DPPH radicals that result in lower reactivity of DPPH radicals. As mentioned in a previous publication, the reaction kinetics of DPPH and ABTS cations are different, which can result in higher values of ABTS assay compared to the values attained by employing DPPH assay [43]. In addition to the variation in peanut variety, most of the previous studies have used ethanol and methanol as a solvent to extract phenolics of peanut sprouts [13,14,35], while in the present study, acetone was applied for the extraction of phenolics. Extraction solvents and extraction methods were also reported to affect the final concentration of phenolics and antioxidant capacities of extracts. It was also mentioned that different solvent has to be applied along with different extraction methods for efficient extraction of phenolic and better assessment of antioxidant capacity [6]. In addition, the reduction of TFC during germination might affect the antioxidant capacities of peanut sprouts since flavonoids contribute significantly towards the antioxidant activities. Many soluble flavonoids such as catechins, isoflavones, and anthocyanin are reported presenting antioxidant capacities. The lower antioxidant capacities of germinated peanuts may be attributed to the decomposition of these flavonoid compounds during the germination period. Additionally, different cultivation methods and different varieties of peanut samples also presented different antioxidant capacities. A similar finding was also reported previously presenting variations in the antioxidant capacities of peanuts of different varieties [44].

### 4.7. Correlation Analyses between Phenolic Content and Antioxidant Activities of Peanut during Germination

As shown in Table S3, the antioxidant capacities of peanut samples were related to their phenolic compounds. For example, the results of TPC values for three varieties of peanut showed a positive and significant correlation (*r* = 0.956, *p* < 0.01) with ABTS values. It is also observed that the TFC values of peanuts also exhibited significant and positive correlation (*p* < 0.01) with DPPH (*r* = 0.964) and FRAP values (*r* = 0.962). Therefore, the lower result of DPPH and FRAP in germinated peanuts may be due to the decrease in TFC values during germination. From Table S3, MAC values found to exhibit a negative correlation (*p* < 0.01) with DPPH (*r* = −0.678) and FRAP (*r* = −0.700) values. The reason behind this finding may be the variation in the trend followed by MAC values and DPPH as well as FRAP values during germination. The MAC values of peanut samples were increased initially and then decreased while the values for antioxidant capacities were decreased first and then increased.

### 4.8. Effects of Germination on Vitamin C Content of Different Varieties of Peanuts

The deficiency of vitamin C causes the scurvy disease is and an essential nutrient for the synthesis of collagen. Vitamin C is also an excellent antioxidant in the food system that helps to retain the active state of several bioactive compounds. In addition, vitamin C is predominantly employed as a marker for nutritional quality in fruits, vegetables along with their processed products [45]. In present study, initially, the vitamin C content of peanut samples was reduced sharply and then increased significantly till the 8^th^ day of germination. Previously, the initial vitamin C content of lupin seeds was mentioned as 1.5 mg/100 g DM and further increased by 866% on the 9th day of germination [46]. Vitamin C was also found to be increased during germination on day 8 in the case of mung bean from 11.69 mg/100 g DW to 285 mg/100 g DW [45]. Although the peanut is also a kind of bean and variation in its vitamin C content is completely different compared to other legumes previously explored. Another study on peanuts has reported a significant decrease in the vitamin C content of peanut sprouts on the 5th day of germination [8]. A similar trend is also observed in Figure 3d. An initial decrease and further increase in vitamin C content in peanut samples were during the germination process. Thus, the peanut sprouts from three different varieties are a rich source of vitamin C. 

### 4.9. Effects of Germination Time on Trans-Resveratrol Content of Different Varieties of Peanuts

Resveratrol is a major phenolic compound present in peanuts and peanut-related foods. It belongs to the stilbene group and is synthesized by the resveratrol synthase. Resveratrol exhibits potent antioxidant, anti-inflammatory, cardioprotective, neuroprotective, glucose, and lipid regulatory properties. Thus, it can protect from several life-threatening diseases such as cancer, liver disease, obesity, diabetes, cardiovascular diseases, Alzheimer’s disease, and Parkinson’s disease [1]. In the present study, the *trans*-resveratrol content of different varieties of peanuts increased with an increase in the germination time. Similar results were also reported in a previous study conducted to develop functional vegetables from peanut sprouts [12]. In the present study, the *trans*-resveratrol content of the peanut variety Silihei was increased drastically from day 4 to day 6 compared to the other two varieties of peanuts (Table 1). The present study also reported different levels of resveratrol content in different varieties of peanut in non-germinated and germinated peanuts [1].

Variations in the *trans*-resveratrol content may be related to the possible variation in the stilbene synthase, fungal invasion, and mechanical damage that may together affect the resveratrol concentration in different varieties of peanuts during germination [47,48].

### 4.10. Effects of Germination on Phenolic Acid Profile of Different Varieties of Peanuts 

In the case of legumes, phenolic acids are mainly concentrated in the seed coat, followed by the original kernel and cotyledons. However, no relationship was observed between TPC, TFC, condensed tannin content, antioxidant activity, and seed coat color of peanuts. Whereas anthocyanin content exhibit a strong correlation with the seed coat color of peanuts [49]. In terms of peanuts, total phenolic acids are mainly concentrated in its skin and hull [37]. Overall, the total content of phenolic acids will increase with the increase in the days of germination. A previous study also mentioned the qualitative and quantitative variation in the phenolic composition depends on the type of legume and germination condition [50].

Phenolic acids are also reported to hinder the growth and development of a crop by inhibiting seed germination due to the inhibition of key enzymes required for seed germination [51]. Among the 10 phenolic acids investigated in the present study (Figure S2c,d and Table 2), some phenolic acids such as gallic acid, *p*-coumaric acid, and ferulic acid were also reported to exhibit strong inhibitory activity on seed germination [52]. *p*-Hydroxybenzoic acid, vanillic acid, and coumaric acid also exhibit a significant impact on peanut germination. This effect is related to the type and concentration of a particular phenolic acid. The variation in the phenolic profile of peanuts during germination is in strong agreement with previous studies on other legumes [50,53].

## 5. Conclusions

Consumer demand for quality food has triggered food scientists to explore novel food products such as peanut sprouts that are appreciated by Chinese societies as a functional vegetables. The moisture content of peanuts followed a “fast-slow-fast” trend during 8 days of germination. The TPC, TFC, and antioxidant capacities of peanuts exhibit a significant decrease after short-term germination followed by a significant increase from the middle of germination. Except for the ABTS value, the antioxidant capacities of germinated peanut samples were lower compared to non-germinated peanuts. The reduction in the antioxidant capacities may be attributed to the loss of TFC as evident from the significant positive correlation between TFC and antioxidant capacities. The variation in the antioxidant capacities by using three different assays may be attributed to the difference in the basic principles of these three methods. Although the TFC content for germinated peanut was lower compared to the raw peanut TPC and MAC values were enhanced during germination. The vitamin C content of peanuts presents a rapid decrease followed by a significant increase with an increase in germination time. A significant increase in the *trans*-resveratrol content was observed in the case of all peanut varieties with an increase in the germination time. Peanut varieties are a rich source of phenolic acids and germinated peanuts present significant variation in the phenolic acids with an increase in the germination time. A significant increment in gallic acid, *p*-coumaric acid, and ferulic acid may inhibit the growth of peanut sprouts. Certain phenolic acids of particular peanut varieties were observed to be reduced with an increase in the germination time. Overall, peanut variety Silihei exhibits the highest amount of resveratrol (8 days), *p*-coumaric acid + syringaldehyde (6 day), ferulic acid (6 day), TPC (8 day), vitamin C (0 day), and ABTS value (8 day) during germination. Whereas the highest amount of gallic acid (8 days), protocatechuic acid (8 days), protocatechualdehyde (2 days), gentisic acid (4 days), chlorogenic acid (8 days), sinapic acid (4 days), TFC (0 days), DPPH value (0 days) and FRAP value (0 days) was observed in Xiaobaisha during germination. The peanut variety Silihong contains the highest amount of *p*-hydroxybenzoic acid (8 days), Syringic acid (0 days), and MAC (4 days) during germination. However, the reason behind their degradation of phytochemicals and the associated mechanism is still unknown. Various tools and techniques of molecular biology can be employed to explore the degradation mechanism of phenolic acids and to further explore the involvement of key genes and key enzymes in the degradation of phenolic acids. In the future, the impact of germination time on other active compounds of germinated peanuts, such as fatty acids needs to be explored.

## Figures and Tables

**Table 1:** *Trans*-Resveratrol content of different kinds of peanuts during sprouting.

Varieties	Germination Time (Days)	*Trans*-Resveratrol (ng/g)
	0	26.52 ± 3.19 ^c^
	2	117.36 ± 11.94 ^c^
**Silihong**	4	468.10 ± 72.17 ^b^
	6	638.51 ± 113.35 ^a^
	8	415.93 ± 27.00 ^b^
	0	19.91 ± 5.57 ^b^
	2	42.64 ± 1.57 ^b^
**Silihei**	4	59.34 ± 5.35 ^b^
	6	487.23 ± 49.29 ^a^
	8	631.14 ± 185.92 ^a^
	0	40.08 ± 2.44 ^d^
	2	52.18 ± 1.85 ^cd^
**Xiaobaisha**	4	119.67 ± 13.82 ^c^
	6	281.74 ± 77.82 ^a^
	8	194.90 ± 43.26 ^b^

Data are expressed as mean ± standard deviation (*n* = 3) on a dry weight basis. Different letters within a row (a–d) represent the statistically significant differences (*p* < 0.05) between the mean values.

**Table 2:** Phenolic acid profile of different peanut samples during germination.

Varieties	Phenolic Acid (μg/g)	Germination Time (Days)				
		0	2	4	6	8
**Silihong**	Gallic acid	4.58 ± 0.38 ^c^	32.15 ± 2.17 ^ab^	25.54 ± 2.49 ^b^	28.99 ± 2.75 ^b^	38.44 ± 3.54 ^a^
	Protocatechuic acid	0.93 ± 0.13 ^cd^	0.53 ± 0.11 ^d^	1.41 ± 0.22 ^bc^	4.23 ± 0.58 ^a^	2.02 ± 0.18 ^b^
	Protocatechualdehyde	3.72 ± 0.23 ^b^	4.50 ± 0.00 ^a^	1.82 ± 0.05 ^c^	0.99 ± 0.15 ^d^	4.01 ± 0.38 ^ab^
	*p*-hydroxybenzoic acid	1.52 ± 0.08 ^b^	2.48 ± 0.23 ^b^	0.74 ± 0.07 ^b^	9.06 ± 2.22 ^a^	9.29 ± 0.02 ^a^
	Gentisic acid	23.95 ± 1.10 ^a^	15.07 ± 1.40 ^b^	13.88 ± 1.19 ^bc^	10.56 ± 1.95 ^c^	11.01 ± 0.68 ^c^
	Chlorogenic acid	19.46 ± 1.69 ^c^	30.80 ± 2.41 ^c^	59.04 ± 3.78 ^c^	105.24 ± 19.93 ^b^	144.43 ± 8.80 ^a^
	Syringic acid	5.87 ± 0.01 ^a^	1.16 ± 0.21 ^c^	0.61 ± 0.21 ^c^	2.46 ± 0.68 ^b^	3.09 ± 0.02 ^b^
	*p*-Coumaric acid + Syringaldehyde	6.82 ± 0.20 ^d^	14.99 ± 1.51 ^c^	19.68 ± 2.23 ^b^	21.02 ± 0.14 ^b^	38.73 ± 2.09 ^a^
	Ferulic acid	1.36 ± 0.21 ^e^	3.51 ± 0.26 ^d^	7.95 ± 0.55 ^b^	6.21 ± 0.34 ^c^	9.47 ± 0.19 ^a^
	Sinapic acid	4.77 ± 0.47 ^b^	6.80 ± 0.48 ^a^	4.96 ± 0.26 ^b^	4.55 ± 0.70 ^b^	6.90 ± 0.12 ^a^
**Silihei**	Gallic acid	5.47 ± 1.60 ^a^	8.13 ± 1.42 ^b^	8.53 ± 2.25 ^b^	13.95 ± 1.31 ^b^	30.87 ± 9.79 ^b^
	Protocatechuic acid	0.66 ± 0.22 ^b^	0.51 ± 0.08 ^b^	0.40 ± 0.16 ^b^	3.71 ± 0.19 ^a^	6.36 ± 0.59 ^a^
	Protocatechualdehyde	4.05 ± 1.00 ^a^	3.76 ± 0.01 ^a^	0.76 ± 0.11 ^b^	0.68 ± 0.21 ^b^	0.71 ± 0.13 ^b^
	*p*-hydroxybenzoic acid	0.36 ± 0.09 ^d^	1.10 ± 0.08 ^c^	1.11 ± 0.26 ^c^	2.09 ± 0.17 ^b^	5.13 ± 0.46 ^a^
	Gentisic acid	26.11 ± 7.21 ^bc^	35.23 ± 2.71 ^ab^	20.30 ± 6.07 ^c^	35.99 ± 4.59 ^ab^	42.48 ± 6.23 ^a^
	Chlorogenic acid	10.52 ± 1.89 ^c^	26.61 ± 10.40 ^c^	19.32 ± 3.80 ^c^	61.45 ± 8.39 ^b^	111.05 ± 11.75 ^a^
	Syringic acid	2.26 ± 0.45 ^a^	0.74 ± 0.10 ^c^	0.63 ± 0.12 ^c^	1.14 ± 0.01 ^bc^	1.71 ± 0.16 ^ab^
	*p*-Coumaric acid + Syringaldehyde	3.84 ± 0.90 ^c^	16.52 ± 1.36 ^bc^	26.21 ± 9.02 ^b^	74.14 ± 8.28 ^a^	60.26 ± 8.17 ^a^
	Ferulic acid	1.67 ± 0.18 ^d^	3.45 ± 0.23 ^cd^	5.92 ± 2.22 ^c^	14.47 ± 1.56 ^a^	10.30 ± 1.20 ^b^
	Sinapic acid	5.55 ± 1.26 ^c^	10.51 ± 2.19 ^ab^	6.46 ± 2.42 ^bc^	12.07 ± 1.55 ^a^	9.82 ± 1.25 ^ab c^
**Xiaobaisha**	Gallic acid	0.66 ± 0.08 ^d^	29.24 ± 0.86 ^b^	24.68 ± 3.15 ^bc^	21.14 ± 1.00 ^c^	39.78 ± 4.44 ^a^
	Protocatechuic acid	1.45 ± 0.44 ^c^	0.69 ± 0.12 ^c^	0.61 ± 0.01 ^c^	5.70 ± 0.51 ^b^	8.20 ± 0.93 ^a^
	Protocatechualdehyde	0.91 ± 0.12 ^c^	4.16 ± 1.03 ^a^	2.99 ± 0.43 ^ab^	2.42 ± 0.32 ^bc^	1.95 ± 0.54 ^bc^
	*p*-hydroxybenzoic acid	2.51 ± 1.02 ^b^	1.26 ± 0.14 ^b^	0.41 ± 0.03 ^b^	5.63 ± 2.16 ^a^	8.31 ± 0.09 ^a^
	Gentisic acid	35.40 ± 9.80 ^a^	39.30 ± 5.03 ^a^	52.96 ± 5.11 ^a^	40.52 ± 4.68 ^a^	39.87 ± 9.24 ^a^
	Chlorogenic acid	19.63 ± 4.97 ^c^	30.38 ± 8.18 ^c^	84.05 ± 6.33 ^b^	106.60 ± 11.84 ^b^	186.65 ± 38.04 ^a^
	Syringic acid	5.31 ± 0.87 ^a^	1.46 ± 0.39 ^b^	0.13 ± 0.03 ^c^	0.83 ± 0.15 ^bc^	1.90 ± 0.26 ^b^
	*p*-Coumaric acid + Syringaldehyde	15.00 ± 0.45 ^c^	9.07 ± 0.94 ^c^	14.28 ± 0.49 ^c^	31.18 ± 3.71 ^b^	53.36 ± 8.63 ^a^
	Ferulic acid	1.16 ± 0.17 ^c^	3.07 ± 0.27 ^b^	5.04 ± 0.28 ^a^	6.15 ± 0.77 ^a^	6.07 ± 0.59 ^a^
	Sinapic acid	15.46 ± 1.67 ^b^	18.50 ± 5.16 ^b^	38.92 ± 5.71 ^a^	28.35 ± 2.15 ^ab^	32.58 ± 8.35 ^a^

Data are expressed as mean ± standard deviation (*n* = 3) on a dry weight basis. Different letters within a row (a–e) represent the statistically significant differences (*p* < 0.05) between the mean values.

## Data Availability

Data is contained within the article and Supplementary Material.

## Supplementary Materials

The following are available online at www.mdpi.com/2076-3921/13/11/2033/s1, Figure S1: Development and length of the radicle of different varieties of peanuts during germination, Figure S2: Typical HPLC chromatograms of standard (a) and sample (b) for *trans*-resveratrol analyses, and standard (c) and sample (d) of phenolic acid analyses. Table S1: Information of peanut samples, Table S2: Moisture content (%) of three varieties of peanut during germination, Table S3: Correlation analysis of antioxidant capacities and phenolic content.

## Abbreviations

**Abbreviation**	**Full Form**
TPC	Total phenolic content
TFC	Total flavonoid content
MAC	Monomeric anthocyanin content
DPPH	2,2-diphenyl-1-picrylhydrazyl
FRAP	Ferric Reducing Antioxidant Power
SK-N-SH	Human Neuroblastoma Cell Line (ATCC HTB-11)
mmol/L	Millimoles per Litre
μg	Microgram
ng	Nanogram
DW	Dry Weight basis
ABTS	2,2′-azino-bis (3-ethylbenzothiazoline-6-sulfonic acid)
TPTZ	2,4,6-tri(2-pyridyl)-s-triazine
TFA	Trifluoroacetic acid
nm	Nanometer
μL	Microliter
GAE	Gallic acid equivalents
CAE	Catechin equivalents
TE	Trolex equivalents
FE^2+^	Fe^2+^ equivalent
HPLC	High-Performance Liquid Chromatography
RP	Reverse Phase
BHT	Butylated hydroxytoluene
PVDF	Polyvinylidene difluoride
LC	Liquid Chromatography
UV	Ultra Violet
IBM	International Business Machines Corporation
SPSS	Statistical Package for the Social Sciences
