# Supplementary material for: Impact of Germination Time on Resveratrol, Phenolic Acids, and Antioxidant Capacities of Different Varieties of Peanut (Arachis hypogaea Linn.) from China
Source: Antioxidants (Basel). 2021 Oct 27;10(11):1714. doi: 10.3390/antiox10111714 (PMC8614862; doi:10.3390/antiox10111714)
Supplement: Supplementary file 1 [file antioxidants-10-01714-s001.zip › antioxidants-1427986-supplementary.pdf]

**Table S1.** Information of peanut samples

| Variety number | Variety name | Type | Place of origin | Year of cropping | Source |
|---|---|---|---|---|---|
| **1** | Silihong | Red peanut | Shandong | 2018 | Laixi Heiyuzhen Co., Ltd. |
| **2** | Silihei | Black peanut | Shandong | 2018 | Laixi Heiyuzhen Co., Ltd. |
| **3** | Xiaobaisha | Light red peanut | Shandong | 2018 | Rizhao Haoyang Agricultural products |

**Table S2.** Moisture content (%) of three varieties of peanut during germination

| Germination time (days) | Silihong (%) | Silihei (%) | Xiaobaisha (%) |
|---|---|---|---|
| 0 | 10.40 ± 1.10 e | 8.73 ± 0.16 e | 10.11% ± 0.81 e |
| 2 | 42.52 ± 0.59 d | 40.17 ± 1.72 d | 39.81% ± 3.69 d |
| 4 | 57.99 ± 2.91 c | 54.03 ± 1.97 c | 49.57% ± 2.80 c |
| 6 | 74.71 ± 4.00 b | 71.10 ± 4.83 b | 61.94% ± 3.39 b |
| 8 | 84.3% ± 2.29 a | 78.97 ± 2.19 a | 77.88% ± 1.58 a |

Data marked with the different letter show significant difference ($p < 0.05$).

**Table S3.** Correlation analysis of antioxidant capacities and phenolic content

| | TPC | TFC | MAC | DPPH | ABTS | FRAP |
|---|---|---|---|---|---|---|
| **TPC** | | 0.273 | -0.328 | 0.193 | 0.956** | 0.427 |
| **TFC** | | | -0.584* | 0.964** | 0.198 | 0.962** |
| **MAC** | | | | -0.678** | -0.311 | -0.700** |
| **DPPH** | | | | | 0.124 | 0.941** |
| **ABTS** | | | | | | 0.360 |
| **FRAP** | | | | | | |

The data were marked with * and **. Sign* indicates significant correlation ($P < 0.05$); ** indicates extremely significant correlation ($p < 0.01$).

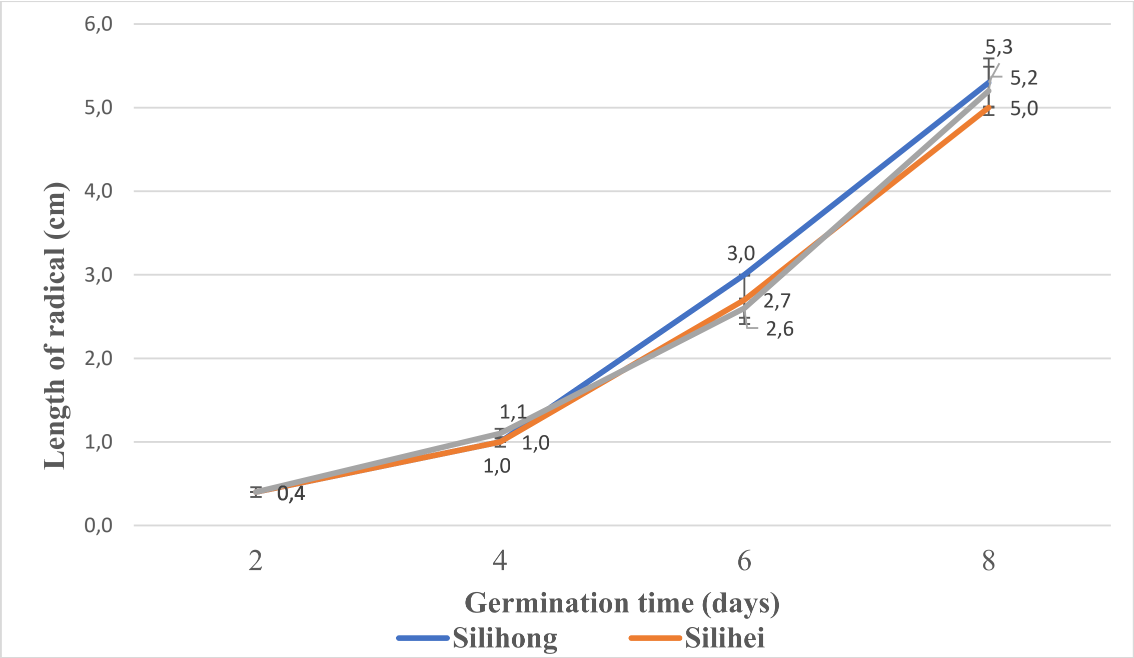


**Figure.** S1**.** Development and length of radicle of different varieties of peanuts during germination

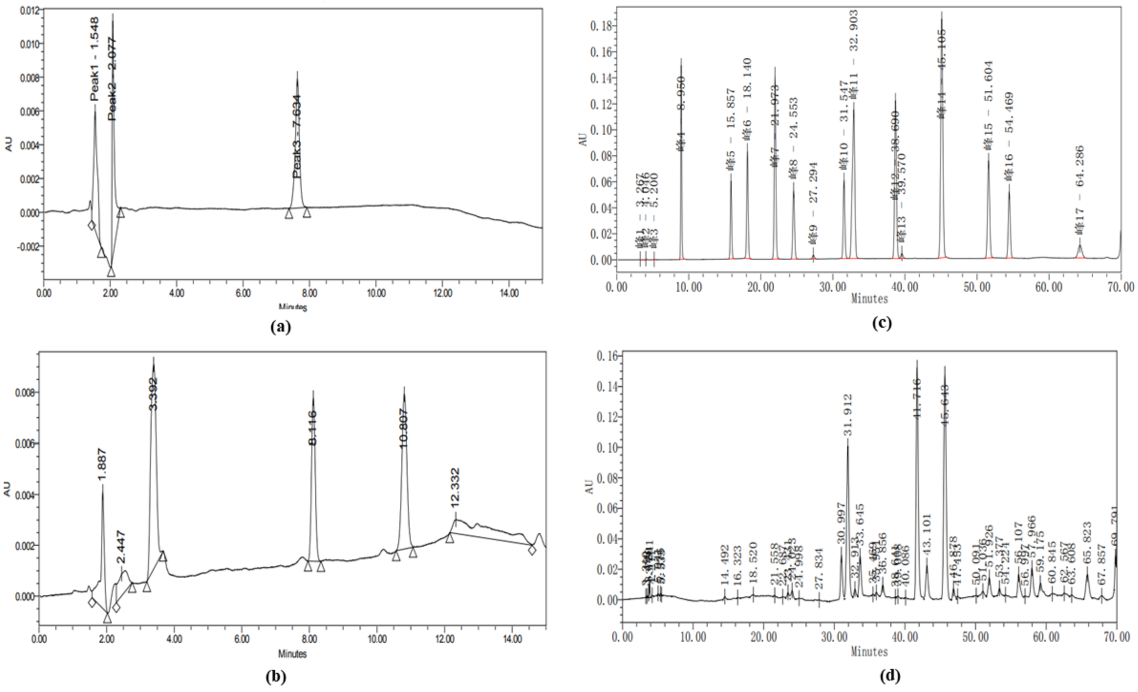


**Supplemental Figure 2.** Typical HPLC chromatograms of standard (a) and sample (b) for *trans*-resveratrol analyses, and standard (c) and sample (d) of phenolic acid analyses.
